# The Role of α7-Nicotinic Acetylcholine Receptors in the Pathophysiology and Treatment of Parkinson’s Disease

**DOI:** 10.3390/ijms26073210

**Published:** 2025-03-30

**Authors:** Eslam ElNebrisi, Yosra Lozon, Murat Oz

**Affiliations:** 1 Department of Biomedical Sciences, Dubai Medical College for Girls, Dubai Medical University, Dubai 20170, United Arab Emirates; 2 Department of Pharmaceutical Sciences, Dubai Pharmacy College for Girls, Dubai Medical University, Dubai 20170, United Arab Emirates; yosra@dmu.ae; 3 Department of Pharmacology and Therapeutics, College of Pharmacy, Kuwait University, Safat 13110, Kuwait

**Keywords:** α7-nicotinic acetylcholine receptor, Parkinson’s disease, neuroprotection, allosteric modulators, L-dopa-induced dyskinesia, neuroinflammation, dopamine release, cholinergic system

## Abstract

The α7 nicotinic acetylcholine receptor (α7-nAChR) is a pivotal regulator of neurotransmission, neuroprotection, and immune modulation in the central nervous system. This review explores its structural and functional attributes, highlighting its therapeutic potential in neurodegenerative disorders, particularly Parkinson’s disease (PD). α7-nAChRs mediate synaptic plasticity, modulate inflammatory responses, and influence dopamine release, positioning them as a promising pharmacological target. Positive allosteric modulators (PAMs) enhance α7-nAChR activity mainly by reducing desensitization, offering a superior therapeutic approach compared with direct agonists. Emerging preclinical studies suggest that α7-nAChR activation mitigates dopaminergic neurodegeneration, improves L-dopa-induced dyskinesia, and reduces neuroinflammation. Despite promising findings, clinical trials have yielded mixed results, necessitating further research into optimizing α7-targeted therapies. This review underscores the significance of α7-nAChRs in PD pathophysiology and highlights future directions for their translational potential in neuroprotection and symptomatic relief.

## 1. Introduction

Acetylcholine (ACh) is one of the most versatile neurotransmitters, regulating numerous physiological processes in both the central and peripheral nervous systems. It plays an important role in cognitive functions, motor control, and autonomic regulation. ACh exerts its effects by interacting with two distinct receptor classes: muscarinic acetylcholine receptors (mAChRs), which are G-protein-coupled receptors, and nicotinic acetylcholine receptors (nAChRs), which are ligand-gated ion channels [1,2].

Nicotinic receptors are further categorized into various subtypes, among which the α7-nAChRs have emerged as a focal point of research [3]. These receptors are distinguished by their high calcium permeability, rapid desensitization, and ability to form homopentameric ion channels. Their widespread expression across critical regions of the central nervous system (CNS), such as the hippocampus, cortex, and basal ganglia, underpins their involvement in key processes, including synaptic plasticity, neuroprotection, and immune modulation.

**Figure 1:**
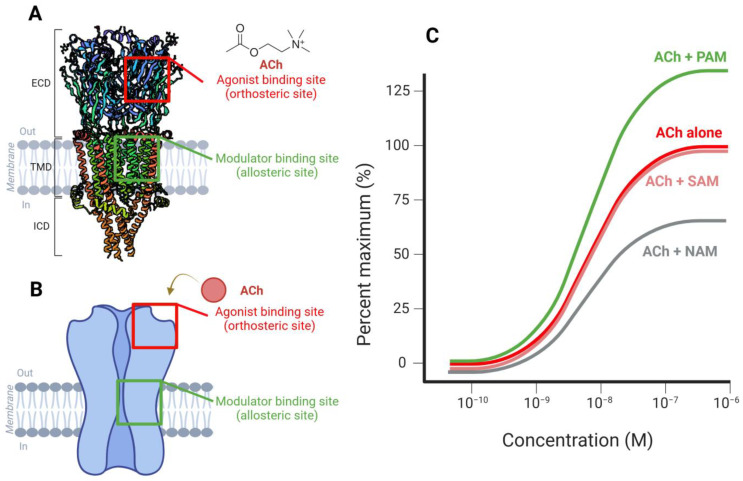
Structural and functional overview of α7 nicotinic acetylcholine receptors (nAChRs) and allosteric modulation. (**A**) Structural representation and Schematic diagram (**B**) of the α7 nAChR, highlighting the two primary ligand-binding sites. The α7 nAChR is homopentameric (each subunit demoted as one color), with each subunit containing an extracellular ligand-binding domain (ECD), four transmembrane domains (TMDs: M1–M4) forming the ion channel, and an intracellular domain (ICD). The orthosteric binding site, where acetylcholine (ACh) and classical agonists bind, is shown in red. The allosteric binding site, where modulators such as positive (PAMs), negative (NAMs), or silent allosteric modulators (SAMs) interact, is marked in green. The receptor spans the lipid bilayer, with an extracellular domain for ligand recognition and a transmembrane region forming the ion channel. (**C**) Concentration–response curves depicting the influence of different modulators on ACh-induced receptor activation. The response to ACh alone (red curve) serves as a baseline. PAMs (green) enhance receptor activity beyond the baseline response, whereas NAMs (gray) reduce receptor activation. SAMs (light red) do not significantly alter receptor activation but may modulate interactions with other compounds. Visuals were created using Biorender with PDB ID for 7EKI [9].

Orthosteric is the term used to describe the binding site for endogenous ligands, such as choline and ACh, and classical agonists binding to the receptor at these sites are therefore referred to as orthosteric agonists [4]. Actions of these orthosteric agonists are effectively blocked by specific antagonists such as Methyllycaconitine (MLA) and α-Bungarotoxin (α-BTX) [5]. In addition, a group of compounds that lack agonist activity on nAChRs and act via a distinct transmembrane binding site is described as allosteric modulators (allo- from the Greek meaning “other”) [6]. Allosteric modulators can influence receptor activity through three primary mechanisms: (1) Positive allosteric modulators (PAMs) can potentiate receptor activity in the presence of an agonist while preserving the spatiotemporal features of synaptic transmission and the receptor’s characteristic rapid desensitization (Type 1) or significantly decreases receptor desensitization and reactivates desensitized receptors (Type 2) [7]; (2) Negative allosteric modulators (NAMs), which inhibit agonist-induced receptor activity or act as open-channel blockers; (3) Silent allosteric modulators (SAMs), which have no direct effect on receptor function but prevent modulation by other allosteric agents (see Figure 1) [8].

These modulatory mechanisms significantly enhance the therapeutic potential of α7-nAChRs by fine-tuning receptor responses [10]. In comparison with conventional α7-nAChR agonists, PAMs have gained prominent relevance as pharmacological agents because of the following advantages: (1) they exhibit greater structural diversity, as the orthosteric site is highly conserved in nAChRs [11]; (2) they provide wider range of final effects; (3) they provide additional neuroprotective potential, as activation of α7-nAChR can be readily limited by desensitization, certain α7-PAMs such as PNU-120596 and AVL-3288 has the ability to maintain conductive state of the receptor and inhibit desensitization [12,13,14,15]. Moreover, neuronal injury has been shown to elevate extracellular choline levels near the site of damage, activating the cholinergic system. The use of PAMs can enhance this injury-induced, α7-dependent neuroprotective mechanism by increasing the efficacy of endogenous choline, thereby reducing the need for high levels of agonist stimulation to achieve neuroprotection [14]. Furthermore, unlike agonists, PAMs do not upregulate α7-nAChRs [16]. In addition, PAMs have higher receptor selectivity than agonists, preserving the temporal properties of endogenous activation and decreasing the tolerance caused by desensitization.

Dysfunction of α7-nAChRs has been implicated in numerous pathologies, including neurodegenerative diseases such as Parkinson’s disease, Alzheimer’s disease, schizophrenia, and autism [5,17,18,19,20,21]. This review delves into the unique structural and functional attributes of α7-nAChRs, highlighting their potential as therapeutic targets for neurodegeneration and inflammatory conditions.

## 2. Structure of α7-nAChRs

The α7-nAChRs are homopentameric ligand-gated ion channels composed of five identical subunits. Each subunit features an extracellular ligand-binding domain (ECD), four transmembrane domains (TMD: M1-M4), and an intracellular domain (ICD) represented as a cytoplasmic loop between M3 and M4 helices (Figure 1A,B). The receptor’s high calcium permeability distinguishes it from other nAChRs, facilitating its critical role in synaptic plasticity and neuroprotection [22,23].

The distribution of nAChRs is relatively conserved across vertebrate species and is not restricted to well-defined brain cholinergic pathways [24,25]. The structure and localization of different nAChR subtypes have been investigated using complementary techniques, including in situ hybridization and PCR to detect specific subunit RNAs, immunoprecipitation for protein subunits, autoradiography, positron emission tomography (PET) and single-photon emission computed tomography (SPECT) imaging, and electrophysiological assays [26,27]. These studies have revealed that α7 is widely distributed in the mammalian brain, with particularly high expression in the hippocampus, hypothalamus, cortex, thalamus, and amygdala, alongside limited expression in the striatum, medulla, and various brain nuclei [19]. This distribution underpins their roles in cognitive processing, motor function, and inflammatory regulation [24,28,29,30,31,32]. Notably, altered distribution and expression patterns of nAChRs have been observed in neurodegenerative conditions, including Parkinson’s disease [33,34]. PET imaging with the radioligand [18F]ASEM, combined with structural magnetic resonance imaging (MRI), was used to assess α7-nAChR distribution in healthy human volunteers. The findings revealed a positive correlation between α7-nAChR distribution and age across multiple brain regions, suggesting increased receptor availability as a compensatory response to declining acetylcholine signaling during aging [35]. Likewise, individuals with mild cognitive impairment exhibited greater availability of α7-nAChR relative to cognitively intact individuals of similar age [36], highlighting the potential role of α7-nAChR as a possible marker for cognitive decline and a potential therapeutic target for age-related and neurodegenerative conditions.

### Genetic Variants and Functional Implications

The α7-nAChRs, encoded by the *CHRNA7* gene on Chromosome 15, are subject to significant genetic variation, including single nucleotide polymorphisms (SNPs). A unique feature of *CHRNA7* is its partial duplication, resulting in the formation of the human-specific gene *CHRFAM7A*. The partially duplicated *CHRFAM7A* acts as a dominant-negative modulator, altering receptor functionality. Through its product, dupα7, integrating with α7 subunits, disrupting receptor assembly and reducing ligand-binding efficiency, leading to downregulated receptor expression and altered signaling capacity [37]. These genetic variants influence susceptibility to neurodegenerative diseases and have been linked to variations in therapeutic responses to α7-targeting agents [38]. However, the functional consequences of these variants remain underexplored, particularly in diverse populations [38,39]. Alterations in cholinergic neurotransmission, whether due to genetic dysregulation or cholinergic denervation, have been implicated in several pathological conditions. Multiple studies have correlated a decline in specific nAChR subtypes, including α7, with Alzheimer’s disease [40], Parkinson’s disease [41,42], and schizophrenia [43]. In Parkinson’s disease, a reduction in α7 receptor expression has been linked to impaired anti-inflammatory signaling and increased susceptibility to neurodegeneration [44], highlighting the importance of preserving or modulating nAChR expression as part of therapeutic strategies for neurodegenerative disorders [3,45].

The functional implications of genetic variations, particularly SNPs, remain underexplored. Specific SNPs in *CHRNA7* and *CHRFAM7A* have been shown to influence receptor expression levels, ligand-binding capacity, downstream signaling, and treatment responsiveness, thereby impacting disease progression and treatment efficacy [38]. For example, *CHRFAM7A* SNPs have been associated with elevated pro-inflammatory cytokine levels, such as tumor necrosis factor-α (TNF-α) and interleukin-1β (IL-1β), suggesting a potential role in regulating inflammatory processes than previously recognized [46,47].

## 3. Parkinson’s Disease: Pathophysiology and Role of α7-nAChRs

PD is a progressive neurodegenerative disorder marked by the selective loss of dopaminergic neurons in the substantia nigra pars compacta, leading to dopamine depletion in the striatum. This disruption manifests as motor symptoms such as bradykinesia, rigidity, resting tremors, and postural instability. Additionally, non-motor symptoms, including cognitive impairment, depression, and sleep disturbances, significantly impair patient quality of life [48,49]. The underlying pathophysiology involves a combination of mitochondrial dysfunction, oxidative stress, neuroinflammation, and protein aggregation. Genetic mutations (e.g., SNCA, LRRK2) and environmental toxins further exacerbate neuronal damage [50,51,52]. As mainly a dysfunction in the dopaminergic system in the brain, Levodopa or L-dopa (L-3,4-dihydroxyphenylalanine) was introduced in the 1960s as a prodrug of dopamine-enhancing intracerebral dopamine concentration. Since its approval by the FDA in 1970, L-dopa has been the gold standard treatment for Parkinson’s disease. However, after several months to years of treatment with L-dopa, patients develop the adverse effects of dyskinesias [53,54,55]. While existing therapies such as L-dopa address motor symptoms, they do not halt disease progression and often lead to complications such as L-dopa-induced dyskinesia (LID) [56].

### 3.1. The Role of Nicotine and α7-Nicotinic Receptors in Neuroprotection

Nicotine, the primary addictive component in tobacco, has garnered attention for its potential neuroprotective effects, particularly through its interaction with α7-nAChRs [57,58,59,60,61,62,63]. Nicotine’s high lipophilicity allows it to cross the blood–brain barrier rapidly, binding to α7-nAChRs and activating pathways that may protect against neurodegeneration [64,65,66].

In the context of PD, studies suggest that nicotine stimulation of α7-nAChRs can mitigate dopaminergic neuron loss, a hallmark of the disease [19]. For instance, the activation of α7-nAChRs has been shown to reduce neuroinflammation by inhibiting astrocyte and microglial activation [64,67]. Additionally, nicotine may enhance the expression of neuroprotective proteins, such as the transcription factor Nurr1, which decreases α-synuclein aggregation, a key pathological feature of PD [68,69]. These mechanisms underpin findings from epidemiological studies, which consistently report a lower incidence of PD among smokers, correlating with the duration and intensity of nicotine exposure [70,71,72].

Furthermore, clinical trials with nicotine patches have demonstrated symptomatic improvements in PD patients, further substantiating its potential role in neuroprotection [73,74,75]. In addition, nicotine administration reduces motor symptoms and mitigates the side effects of L-dopa therapy [76], such as dyskinesia, by modulating dopamine release through α7-nAChR activity [77].

While nicotine’s interaction with α7-nAChRs offers promising insights into neuroprotective strategies, it is critical to decouple these benefits from the harmful effects of smoking. Selective α7-nAChR agonists or positive allosteric modulators represent a promising avenue for harnessing these neuroprotective effects without the detrimental consequences of nicotine addiction [78].

### 3.2. Role of α7-nAChRs in PD

#### 3.2.1. Dopaminergic and Cholinergic Systems Correlation and Dopamine Release

Dopamine inputs to the striatum originate primarily from midbrain dopaminergic neurons located in the ventral tegmental area (VTA) and the substantia nigra pars compacta (SNpc). These projections target both the ventral (nucleus accumbens) and dorsal striatum (caudate–putamen). The striatum is densely innervated by axonal varicosities of dopaminergic neurons, forming dopaminergic synapses that modulate motor and reward functions [79,80].

**Figure 2:**
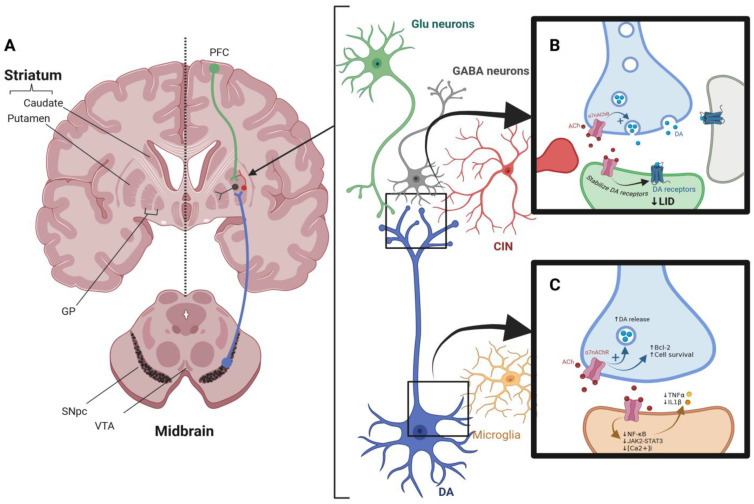
Mechanistic Role of α7 Nicotinic Acetylcholine Receptors (α7-nAChRs) in Dopaminergic Regulation, Neuroinflammation, and LID in Parkinson’s Disease. (**A**) Schematic representation of the nigrostriatal dopaminergic pathway. Dopaminergic (DA) neurons originating from the substantia nigra pars compacta (SNpc), beside the ventral tegmental area (VTA), project to the striatum, modulating motor control. Within the striatum, dopaminergic signaling is regulated by cholinergic interneurons (CINs), glutamatergic (Glu) inputs from the prefrontal cortex (PFC), and GABAergic neurons projecting to globus pallidus GP. (**B**) Role of α7-nAChRs in LID modulation. The activation of α7-nAChRs on presynaptic terminals enhances dopamine (DA) release and stabilizes DA receptor function, thereby reducing LID severity. (**C**) Neuroprotective and anti-inflammatory effects of α7-nAChRs in microglia. Activation of α7-nAChRs suppresses the release of pro-inflammatory cytokines (TNF-α, IL-1β) by inhibiting key inflammatory pathways (NF-κB, JAK2-STAT3, and intracellular calcium signaling), enhancing DA neuronal survival via Bcl-2 up-regulation, supporting neuron health in PD brain.

Cholinergic interneurons (CINs) (see Figure 2A) in the striatum represent the primary source of cholinergic input. These interneurons express both muscarinic and nicotinic acetylcholine receptors, playing a crucial role in modulating dopaminergic signaling. While muscarinic receptors mediate slower, modulatory effects, nicotinic receptors facilitate rapid synaptic transmission, directly influencing dopamine release [81,82]. Nicotinic receptors are critical regulators of neuronal excitability and neurotransmitter release in the CNS. Presynaptic nAChRs enhance dopamine release by modulating vesicular dynamics, while postsynaptic nAChRs contribute to excitatory synaptic inputs. In the hippocampus, striatum, and subcortical regions, nAChRs are integral to maintaining synaptic plasticity and neurotransmitter homeostasis [83].

Studies using animal models have highlighted the role of nAChRs in dopamine modulation [84,85]. Blaha and Winn demonstrated that nicotine administration increased dopamine efflux in the VTA and SN. This effect was significantly attenuated following lesioning of the VTA, underscoring the importance of cholinergic innervation in modulating dopamine pathways [86,87,88]. Similarly, Forster and Blaha reported that electrical stimulation of the VTA enhanced dopamine release via nicotinic and glutamatergic receptors in the striatum [89]. Quik et al. extended these findings by investigating the effects of nicotine treatment in MPTP-lesioned primates [90]. Their results revealed increased levels of tyrosine hydroxylase, dopamine transporter, and vesicular monoamine transporter in nicotine-treated animals compared with untreated controls. These changes were accompanied by elevated striatal dopamine levels, indicating that nicotine can ameliorate dopaminergic deficits in Parkinsonian models [91]. Quarta et al. examined the role of α7-nAChRs in dopamine release using mutant mice lacking these receptors [92] and demonstrated a significant reduction in nicotine-stimulated dopamine release in the absence of α7-nAChRs. Moreover, the application of selective α7-agonists such as choline restored dopamine release, emphasizing the receptor’s role in dopaminergic signaling. Similar studies using fast-scan cyclic voltammetry have confirmed that dopamine release is predominantly mediated by nicotinic, rather than muscarinic, acetylcholine receptors [93]. Additionally, research on induced pluripotent stem cells (iPSCs) derived from Parkinson’s disease patients carrying the LRRK2 G2019S mutation has shown that this mutation disrupts the membrane localization of dopamine D3 receptors (D3R) and nicotinic acetylcholine receptors (nAChR), impairing their heteromeric formation, which is crucial for neuronal homeostasis. Normalizing LRRK2 activity was found to restore D3R-nAChR heteromeric localization and function, highlighting a potential therapeutic target for preserving dopaminergic neuron integrity [94]. A summary of α7-nAChRs modulatory effects on dopaminergic neurons is shown in Figure 2B.

#### 3.2.2. Immune Modulation via Nicotinic Receptors

Activation of the immune system in the CNS occurs in response to various insults, including stroke, neurodegenerative diseases, spinal cord injury, multiple sclerosis, and traumatic brain injury. The innate immune system within the CNS is primarily represented by microglia, the resident macrophages of the brain. Under physiological conditions, microglia exhibit a resting phenotype characterized by a small cell body and highly ramified processes [95]. These cells play critical roles in maintaining CNS homeostasis, including the phagocytosis of apoptotic cells and debris, synaptic pruning, and the secretion of neurotrophic factors such as insulin-like growth factor-1 (IGF-1) and transforming growth factor-β (TGF-β). However, chronic activation of microglia in response to injury or pathogen invasion can lead to the release of pro-inflammatory cytokines and reactive oxygen species (ROS), exacerbating neuronal damage and contributing to the pathogenesis of neurodegenerative diseases such as PD [96].

Wang et al. were the first to demonstrate the interaction between the cholinergic and immune systems [97]. Further studies by Shytle et al. confirmed the expression of α7-nAChRs on microglia and their critical role in attenuating lipopolysaccharide (LPS)-induced TNF-α release [97,98,99,100]. Nicotine has been shown to decrease microglial activation and protect neurons by reducing oxidative and inflammatory stress [101]. Activated microglia express various surface receptors that mediate immune responses, including Toll-like receptors (TLRs) and nAChRs [102]. Studies have demonstrated that persistent microglial activation is associated with elevated levels of cytokines such as TNF-α, IL-1β, and interferon-γ (IFN-γ) [103]. These pro-inflammatory mediators contribute to neurodegeneration by inducing oxidative stress, mitochondrial dysfunction, and neuronal apoptosis [104]. In PD, neuroinflammation in the substantia nigra is a hallmark of disease progression, highlighting the importance of modulating microglial activity to protect dopaminergic neurons [105,106]. In addition to microglia, α7-nAChRs are expressed on astrocytes, where they modulate neuroinflammation and support neuronal survival. The activation of α7-nAChRs on astrocytes inhibits hydrogen peroxide (H2O2)-induced apoptosis by maintaining mitochondrial membrane potential and regulating the Bax/Bcl-2 ratio [107,108]. Liu et al. demonstrated that this protective effect is abolished in the presence of methyllycaconitine, an α7-nAChR antagonist, underscoring the receptor’s role in astrocyte functions [108,109,110].

The anti-inflammatory effects of α7-nAChRs are mediated through both calcium-dependent and calcium-independent pathways [17,111]. The activation of these receptors inhibits nuclear factor-kappa B (NF-κB) signaling and reduces the expression of inducible nitric oxide synthase (iNOS) and cyclooxygenase-2 (COX-2) [112]. Moreover, α7-nAChRs activate the Janus kinase 2 (JAK2)-STAT3 signaling cascade, promoting the expression of anti-inflammatory genes. A summary of α7-nAChRs immunomodulatory effects is depicted in Figure 2C. These mechanisms highlight the therapeutic potential of α7-nAChR agonists in reducing neuroinflammation and protecting against neurodegeneration [64]. These preclinical studies suggest that selective α7-agonists and PAMs can reduce microglial activation, enhance neuronal survival, and improve behavioral outcomes in animal models of PD.

#### 3.2.3. Effect of Nicotinic Receptors on L-Dopa-Induced Dyskinesia

L-Dopa-induced dyskinesia (LID) is a debilitating complication of prolonged L-Dopa therapy in PD. It manifests as abnormal involuntary movements (AIMs) that severely impair the quality of life for patients [56]. The pathophysiology of LID is multifactorial, involving presynaptic dysregulation of dopamine metabolism and postsynaptic hypersensitization of dopamine receptors. Chronic L-Dopa use leads to maladaptive synaptic plasticity and aberrant corticostriatal signaling, exacerbating motor dysfunction [113]. Despite its unparalleled efficacy in managing motor symptoms, L-Dopa necessitates adjunctive strategies to address LID without compromising therapeutic outcomes. Current pharmacological approaches, including amantadine, offer some relief by antagonizing NMDA receptors, yet their effects are often transient and accompanied by adverse events such as hallucinations and edema [114]. Surgical options such as deep brain stimulation (DBS) provide effective symptom control for refractory LID but are invasive and require rigorous patient selection because of the associated surgical risks [115]. These challenges underscore the critical need for novel, non-invasive therapeutic interventions.

Recent studies have highlighted the therapeutic potential of α7-nAChRs in addressing LID. The activation of α7-nAChRs regulates dopamine receptor sensitivity and intracellular signaling, thereby mitigating postsynaptic receptor hypersensitization [65]. Additionally, presynaptic α7-nAChRs influence dopamine release by modulating vesicular dynamics and stabilizing synaptic transmission [19,65,116] (Figure 2B). The dual role of α7-nAChRs in presynaptic and postsynaptic modulation provides a robust framework for reducing the maladaptive neuronal activity underlying LID.

Preclinical evidence further supports the efficacy of α7-nAChRs in ameliorating LID. For instance, long-term nicotine administration in MPTP-lesioned primates and 6-OHDA rodent models has demonstrated a significant reduction in dyskinesia severity, with effects attributed to the activation of α7 and α4β2*/α6β2* nAChRs [117,118]. These findings have been corroborated by knockout studies, which highlight the necessity of α7-nAChRs in mediating the anti-dyskinetic effects of nicotinic agonists [19]. Furthermore, pharmacological agents such as ABT-126, a selective α7 nAChR agonist, have shown dose-dependent efficacy in reducing AIMs in MPTP-treated monkeys [119,120,121]. The neuroprotective effects of ABT-126 are achieved through the mitigation of oxidative stress, modulation of inflammatory pathways, and attenuation of receptor hypersensitivity [122]. Clinical trials investigating ABT-126 in Alzheimer’s disease have demonstrated its safety and tolerability, providing a strong foundation for its application in PD-related dyskinesia [123,124].

Translational studies further underscore the potential of α7-nAChRs in LID management. Nicotine patches, for example, have been explored for their ability to reduce dyskinesia severity in PD patients [125]. Preliminary findings suggest that transdermal nicotine offers a non-invasive means of harnessing α7-nAChR activation to improve motor control. However, long-term adherence and safety profiles require further evaluation. Mechanistically, the anti-dyskinetic effects of α7-nAChRs extend beyond dopaminergic modulation. The activation of these receptors reduces maladaptive corticostriatal plasticity and inhibits neuroinflammatory processes by downregulating pro-inflammatory cytokines such as TNF-α and IL-1β [126,127]. Additionally, α7-nAChRs modulate intracellular signaling cascades, including the JAK2-STAT3 and PI3K-Akt pathways, which enhance neuronal survival and synaptic transmission [128,129]. These multifaceted actions position α7-nAChRs as a central therapeutic target for addressing the complex pathophysiology of LID [130].

### 3.3. Receptor Cross-Talk and Systems-Level Integration

α7-nAChRs operate within a highly interconnected neurochemical environment, and their function is shaped by dynamic cross-talk with other receptor systems. Understanding these interactions is critical for developing more precise and synergistic therapeutic strategies for PD.

α4β2-nAChRs are among the most widely studied nicotinic subtypes. These heteromeric receptors are densely expressed in the nigrostriatal pathway and play a crucial role in modulating dopamine release [131]. While α7-nAChRs are primarily involved in calcium signaling and anti-inflammatory pathways, α4β2 receptors influence vesicular dopamine dynamics and motor regulation. Studies show that nicotine’s therapeutic effect on LID involves both receptor subtypes, with cross-antagonism leading to diminished benefit. Co-targeting α7 and α4β2 may, therefore, yield additive or synergistic effects [117].

Similarly, α6-containing nAChRs (particularly α6β2β3*), which are highly localized to dopaminergic terminals in the substantia nigra and striatum, regulate dopamine release and are highly vulnerable in PD [132,133]. Cross-talk between α6 and α7 receptors has been suggested, particularly in dopaminergic neurons where both may coordinate release and survival signaling. Loss of α6-containing nAChRs in PD further supports the rationale for α7-nAChR upregulation as a compensatory neuroprotective mechanism [132,134].

Glutamate receptors, including NMDA and AMPA subtypes, also interact with α7-nAChRs. The activation of α7-nAChRs enhances glutamate release and potentiates NMDA receptor function, contributing to long-term potentiation (LTP) and synaptic plasticity [30]. This interaction is double-edged; while it supports cognitive function and motor learning, it may also promote excitotoxicity in pathological states. This emphasizes the need for fine-tuned modulation of α7-nAChRs, particularly in combination with glutamatergic-targeting agents [2].

GABAergic receptors are also influenced by α7-nAChRs. Studies have shown that α7 activation modulates inhibitory interneuron activity, indirectly regulating the balance between excitation and inhibition in basal ganglia circuits [135,136,137]. In hippocampal networks, α7-nAChRs located on interneurons enhance GABA release, affecting oscillatory activity relevant to cognition. This interaction may have therapeutic implications for managing PD-associated cognitive and affective symptoms [138,139].

In addition, 5-HT3 serotonin receptors, which are similar to nAChRs, are ligand-gated ion channels, share structural similarities, and are often co-expressed with α7-nAChRs. Both influence emesis, cognition, and reward pathways [140]. Cross-talk between these systems could modulate synaptic responsiveness to serotonergic tone, especially in brainstem and limbic regions [141,142].

Finally, interaction with dopaminergic receptors, particularly D3 receptors, is highly relevant in PD. Studies using iPSC-derived neurons from PD patients carrying LRRK2 mutations demonstrate disrupted D3R–α7-nAChR complex formation, impairing synaptic signaling, and homeostasis [94]. This suggests that α7-nAChR-based therapies could help restore receptor interplay critical for dopaminergic balance [142].

Taken together, these examples underscore the necessity of considering receptor cross-regulation, compensatory signaling, and co-expression profiles in drug design. Targeting α7-nAChRs in combination with other systems—either pharmacologically or through precision-medicine approaches—may enhance therapeutic efficacy, minimize side effects, and address both motor and non-motor symptoms of Parkinson’s disease.

## 4. Preclinical Evidence

Several α7-nAChR-targeting compounds have demonstrated neuroprotective and anti-inflammatory effects in animal models of PD [64]. PNU-282987, an α7-nAChR agonist, has been examined in a 6-hydroxydopamine (6-OHDA) rat model of PD. In this study, PNU-282987 improved motor deficits, mitigated tyrosine hydroxylase loss in the substantia nigra (SN), and modulated immune responses (suppressed astrocyte overactivation, enhanced the number of regulatory T cells and associated inflammatory cytokines), suggesting its neuroprotective and anti-inflammatory potential. Additionally, it significantly upregulated the expression of α7-nAChR in 6-OHDA-lesioned rats [143]. These findings suggest PNU-282987’s potential to modulate neuroinflammation and promote neuroprotection in Parkinson’s disease models. Similarly, (E)-nicotinaldehyde O-cinnamyloxime, a nicotine analog, has demonstrated neuroprotective effects by improving cell viability and reducing oxidative stress in an in vitro model of PD [144,145]. This compound was shown to mitigate rotenone-induced neuronal toxicity in SH-SY5Y cells, suggesting its potential therapeutic role in PD models [145]. In addition, PHA 543613 (6 mg/kg) has partially restored striatal dopamine transporter (DAT) density in a 6-OHDA lesioned rat model and reduced neuroinflammation, as measured by TSPO (a microglial activation marker) density, compared with vehicle-treated controls [146]. Another compound, GTS-21 (DMXBA), an α7-nAChR agonist, has restored motor activity, prevented dopaminergic neuronal loss, and inhibited microglial activation and pro-inflammatory gene expression in MPTP-induced PD models [101,147]. Moreover, α7-nAChR agonists such as ABT-126 and ABT-107 have been reported to reduce LID in Parkinsonian non-human primates. These compounds demonstrated anti-dyskinetic effects without exacerbating parkinsonian motor deficits, further supporting the therapeutic potential of α7-nAChR activation in PD [148,149].

Several pharmacological agents targeting α7-nAChRs have demonstrated significant therapeutic potential in PD by enhancing cholinergic signaling, reducing neuroinflammation, and modulating dopaminergic neurotransmission. These agents include orthosteric agonists such as PNU-282987 and PHA 543613, which bind directly to the receptor’s active site, leading to calcium influx and attenuation of microglial activation through anti-apoptotic and anti-inflammatory pathways [150]. Other direct agonists, including ABT-126 and GTS-21 (DMXBA), have shown efficacy in improving motor and cognitive symptoms and reducing LID via neuroprotective mechanisms, as discussed earlier [148,149]. In addition, PAMs such as PNU-120596, a Type II PAM, bind to allosteric sites on α7-nAChRs to stabilize the receptor in its open state and reduce desensitization [7]. This potentiates the effect of endogenous choline, allowing prolonged receptor activation without upregulation. Natural compounds such as curcumin and apigenin have also emerged as promising PAMs, demonstrating antioxidant, anti-inflammatory, and cholinergic-enhancing effects relevant to PD pathogenesis [151,152]. Conversely, antagonists such as MLA and α-BTX bind to the orthosteric site and inhibit receptor activation. A schematic overview of these mechanisms is presented in Figure 1, illustrating receptor binding sites and downstream effects, including increased calcium influx, suppression of pro-inflammatory cytokines (e.g., TNF-α, IL-1β), enhanced dopamine release, and reduction in LID severity.

Ongoing research is increasingly exploring phytochemicals and synthetic analogs as novel modulators of α7-nAChRs, aiming to improve selectivity, reduce side effects, and enhance brain penetrance. For example, curcumin has been classified as a PAM II and has been shown to significantly reduce receptor desensitization and support neuroprotection in preclinical models of neuroinflammation and PD [151,153,154]. Similarly, apigenin, a dietary flavonoid, has been shown to enhance acetylcholine-induced calcium responses and reduce oxidative stress, making it a candidate for future clinical development [155]. These natural compounds represent a valuable avenue for future research, particularly in combination therapy strategies or as templates for designing next-generation PAMs with improved pharmacological profiles.

While these preclinical findings are promising, it is important to acknowledge that the applied PD models, including toxin-based (e.g., MPTP, 6-OHDA) and in vitro systems, only partially represent the complexity of human Parkinson’s disease. These may not demonstrate the progressive, multisystem nature of the disorder. Therefore, in a state of transition from preclinical to clinical investigations, results should be interpreted with caution, considering these limitations in study design and expectations. Collectively, these results strengthen the growing evidence supporting α7-nAChR modulators as promising therapeutic agents for PD.

## 5. Current Clinical Therapeutic Status

While preclinical findings are promising, clinical trials specifically targeting α7-nAChR agonists in PD patients are limited. A phase 2 randomized, placebo-controlled clinical trial aimed to examine AQW051, an α7-nAChR agonist, effects on LID and motor and non-motor symptoms in PD patients [156]. Although the study did not meet its primary endpoints, exploratory findings suggested potential pro-cognitive effects. Several factors may have contributed to the limited efficacy, including suboptimal dose selection, short trial duration, clinical heterogeneity among participants, and the absence of biomarker-guided stratification. These issues highlight the need for a more refined trial design to assess the therapeutic potential of α7-nAChR modulation in PD fully. In general, the neuroprotective and anti-inflammatory properties observed in animal models provide a strong rationale for further clinical investigation. A phase 2a randomized, placebo-controlled trial was planned to evaluate AZD0328, a selective α7 nicotinic receptor agonist, for its potential to improve cognitive outcomes in patients with Parkinson’s disease with mild cognitive impairment; however, the trial was withdrawn prior to enrollment because of logistical and COVID-19 pandemic-related delays [157]. A summary of the clinical status of these two candidates is presented in the table below (Table 1).

## 6. Challenges and Future Directions

Several key challenges are required to be addressed to enable the translating of α7-nAChR modulators into effective therapies for Parkinson’s disease. One of the critical priorities is optimizing drug delivery to the CNS to ensure adequate target activity and minimizing off-target effects through improved pharmacokinetics and receptor selectivity. Personalized treatment approaches could address variability in patient responses because of genetic differences in *CHRNA7* and *CHRFAM7A* expression. In addition, large-scale clinical trials are essential to establish the long-term efficacy and safety profile of these therapies, and future research needs to investigate the development of biomarkers as a tool to stratify patients who are most likely to benefit from α7-nAChR-targeting interventions.

In this context, genetic variants in CHRNA7 and CHRFAM7A offer promising opportunities for biomarker development and patient stratification in future clinical trials targeting α7-nAChRs [158]. For instance, specific single nucleotide polymorphisms (SNPs) within CHRFAM7A—such as the 2-bp deletion polymorphism (rs67158670)—have been associated with heightened pro-inflammatory cytokine release, including TNF-α and IL-1β, in response to immune challenges [46]. Individuals carrying these polymorphisms may exhibit diminished anti-inflammatory responses to α7-nAChR agonists or PAMs, potentially explaining variability in therapeutic outcomes observed in clinical studies [159]. Moreover, expression levels of CHRFAM7A and CHRNA7 vary significantly across populations and individuals, influencing receptor availability and functional responses [37,46]. Incorporating genotyping into clinical trial enrollment could allow for stratification of participants based on predicted receptor function or inflammatory reactivity, enabling more targeted interventions and reducing trial heterogeneity. This personalized approach may also guide dose adjustments, choice of compound (agonist vs. PAM), and even prediction of adverse effects, thus enhancing the clinical utility and translational success of α7-nAChR-targeted therapies in neurodegenerative diseases.

Furthermore, exploring combination therapies that integrate α7-nAChR agonists or modulators with existing PD treatments, such as dopaminergic agents, could yield synergistic benefits. Despite these challenges, a growing body of evidence supports the therapeutic potential of α7-nAChRs modulation in alleviating LID and other PD-related symptoms, offering hope for meaningful advances in patient care.

## 7. Conclusions

The α7-nicotinic acetylcholine receptor represents an important pharmacological target for modulating neurodegeneration, synaptic plasticity, and immune responses in Parkinson’s disease. Its widespread expression and ability to influence dopamine release and inflammatory pathways underscore its therapeutic relevance. Positive allosteric modulators (PAMs) offer significant advantages over traditional agonists by enhancing receptor activity while minimizing desensitization. Preclinical findings strongly support the role of α7-nAChRs in mitigating neuroinflammation and improving L-dopa-induced dyskinesia, yet clinical translation remains a challenge. Future research should focus on optimizing pharmacokinetics, personalizing treatments based on genetic variations, and exploring combination therapies to maximize clinical benefits. Overcoming these hurdles could establish α7-nAChR modulators as transformative agents in Parkinson’s disease management, providing both neuroprotective and symptomatic relief.

## Figures and Tables

**Table 1:** Summary of α7-nAChR drug candidates in clinical trials for PD-related indications.

Drug Candidate	Developer	Target	Clinical Phase	PD Indication Tested	Trail Status	Notes
AQW051	Novartis	Partial α7-nAChR agonist	Completed Phase 2a	Motor symptoms and LID in PD	Completed	Evaluated in PD patients; well tolerated but showed no consistent efficacy [156]
AZD0328	AstraZeneca	Selective α7-nAChR agonist	Planned Phase 2a	Parkinson’s disease with mild cognitive impairment	Withdraw before enrolment	Trial NCT04810104; planned but not initiated due to COVID-19 delays [157]

## Data Availability

The original contributions presented in the study are included in the article; further inquiries can be directed to the corresponding author/s.

## Abbreviations

The following abbreviations are used in this manuscript:

ACh	Acetylcholine
α7 nAChR	α7 nicotinic acetylcholine receptor
6-OHDA	6-hydroxydopamine
LID	L-dopa-induced dyskinesia
mAChRs	muscarinic acetylcholine receptors
MLA	methyllycaconitine
NAMs	Negative allosteric modulators
nAChRs	nicotinic acetylcholine receptors
PAMs	Positive allosteric modulators
PD	Parkinson’s disease
SAMs	Silent allosteric modulators
